# Using SARS-CoV-2 Sequencing Data to Identify Reinfection Cases in the Global Emerging Infections Surveillance Program, United States

**DOI:** 10.3201/eid3014.240231

**Published:** 2024-11

**Authors:** Deanna Muehleman, Bill Gruner, Vivian Hogan, Padraic Fanning, Carol Garrett, Jennifer Meyer, Kelsey Lanter, Sarah Purves, Laurie DeMarcus, Jeffrey Thervil, Bismark Kwaah, Paul Sjoberg, Elizabeth Macias, Anthony Fries

**Affiliations:** US Air Force School of Aerospace Medicine and Defense Centers for Public Health, Dayton, Ohio, USA (D. Muehleman, B. Gruner, V. Hogan, P. Fanning, C. Garrett, J. Meyer, K. Lanter, S. Purves, L. Demarcus, J. Thervil, B. Kwaah, P. Sjoberg, E. Macias, A. Fries); JYG Innovations, Dayton (D. Muehleman, B. Gruner, S. Purves); Henry Jackson Foundation, Rockville, MD, USA (V. Hogan); ERP360 Solutions Group LLC, Washington, DC, USA (P. Fanning, J. Meyer, K. Lanter); Innovative Element LLC, Washington (L. Demarcus, J. Thervil, B. Kwaah, P. Sjoberg)

**Keywords:** COVID-19, respiratory infections, severe acute respiratory syndrome coronavirus 2, SARS-CoV-2, SARS, coronavirus disease, zoonoses, viruses, coronavirus, reinfection, whole-genome sequencing, public health surveillance, United States

## Abstract

The Centers for Disease Control and Prevention defines SARS-CoV-2 reinfection as a positive COVID-19 test result >90 days after the collection date for the initial positive test or if sequencing confirms a different lineage is causing the reinfection. Reinfection dynamics have been examined by using PCR or antigen surveillance data. We identified patients in the US Military Health System who had >1 positive SARS-CoV-2 test during March 2020–July 2022 by using whole-genome sequencing data to identify reinfection cases, then compared those data with patient demographics, symptoms, and vaccination status. We identified 267 reinfections, of which 90% were caused by the SARS-CoV-2 Omicron variant. Reinfection symptom severity correlated with initial symptom severity and time since first infection. Furthermore, we found intrahost mutation rates varied greatly in 72 cases of continuing infections with the same variant. Continued investigations of reinfections caused by emerging SARS-CoV-2 variants of concern is needed to maintain US military readiness.

Most of the human population in the United States has been infected >1 time with SARS-CoV-2 (*1*); much of that exposure occurred during the emergence of the SARS-CoV-2 Omicron variant. As the Omicron variant emerged globally in November 2021 and was first reported in the United States in December 2021 (*2*), the frequency of reinfections also increased (*3*). The Centers for Disease Control and Prevention (CDC) defines a reinfection as a positive COVID-19 test result >90 days after the initial positive test date (*4*). Reinfections have been examined by using PCR or antigen testing (*3*,*5*), and those studies used the >90-day definition. In addition, some studies have used genomic sequencing to define reinfections (*6*–*9*); meta-analyses have been performed in some of those works (*9*–*11*). Others have defined reinfections by using the rate of single-nucleotide variant (SNV) accumulation and have compared those rates with expected rates of mutation (e.g., 1 SNV/2 weeks) (*7*,*8*,*12*). However, reinfection dynamics might be influenced by the infecting SARS-CoV-2 variant; as few as 7 days between Omicron variant reinfection have been reported (*6*).

The Omicron variant has shown a remarkable ability to evade both vaccine-derived immune responses and those from prior infections (*3*,*13*), and waning immunity can occur faster for the Omicron variant than other variants (*9*–*11*). Hybrid immunity from antigen exposure through previous infection plus vaccination might provide better protection against the Omicron variant than infection or vaccination alone, but to a lesser extent than for other variants (*9*–*11*). Since the Omicron variant emerged and a greater understanding of different SARS-CoV-2 variants has evolved (*14*), it is crucial to continue investigating reinfection dynamics.

Because of the variability of host immune responses to SARS-CoV-2, a single reinfection phenotype or outcome likely does not exist (*15*). Vaccine-derived neutralizing antibodies decrease over time and do not completely prevent infection (*16*), and antibody titers wane after infection as well (*17*). In addition, time intervals between exposures to different SARS-CoV-2 antigens can influence the breadth of the immune response (*18*).

We used retrospective clinical testing and sequencing data from public health surveillance specimens to characterize the dynamics of reinfections in the Military Health System (MHS), leveraging the activities of the Department of Defense (DoD) Global Respiratory Pathogen Surveillance Program (GRPSP). We analyzed continuing infections (the same virus clade at 2 collection timepoints) and reinfections (different clades at the first and second collections timepoints). We collected demographic and symptoms data from persons who had reinfections determined by using whole-genome sequencing. Furthermore, we identified continuing infections, for which longitudinal specimens were collected, and analyzed genetic variations in those putative continuing infections over time. We conducted this study under a not research determination according to the Air Force Research Laboratory Institutional Review Board (protocol no. FWR20220269N).

## Methods

### Data Collection

This study encompasses the beginning of the COVID-19 pandemic through the emergence of the Omicron BA.5 variant (March 2020–July 2022). The DoDGRPSP is a global program that characterizes respiratory infections in US military service members and military healthcare beneficiaries (*19*,*20*). We used 2 approaches to capture reinfection specimens. First, a primary function of the program is to collect and test specimens weekly from a random set of 6–10 patients manifesting influenza-like illness at each of >100 DoD treatment facilities globally. Influenza-like illness is defined as a fever (>38°C) and cough or sore throat; or fever accompanied by >2 symptoms associated with influenza or COVID-19; or a physician-diagnosed influenza-like illness (*20*). Each influenza-like illness encounter includes a patient questionnaire that collects demographic (sex, age, and location), symptomatic (onset, temperature/fever, cough, sore throat, fatigue, aches, chills, headache, dyspnea, loss of taste/smell, nausea, vomiting, and diarrhea), and vaccination information. In cases where questionnaires were not available or incomplete, we used codes from the International Classification of Diseases, 10th Revision, for symptoms obtained from MHS Data Repository records. Second, an additional activity of the DoDGRPSP is routine sequencing of residual clinical specimens from throughout the MHS that are positive for SARS-CoV-2, influenza, or other respiratory pathogens. Because of the unlikelihood of identifying reinfections from random encounters characterized in the influenza-like illness program alone, we augmented our dataset by including convenience samples of SARS-CoV-2–positive specimens tested at the US Air Force School of Aerospace Medicine epidemiology laboratory. We combined genotypic data from both initiatives to identify as many reinfection cases as possible.

To quantify severity, we used questionnaire and MHS Data Repository data for hospitalization, ventilation, and specificity of care. We slightly modified symptom severity indexes according to the codes from the International Classification of Diseases, 10th Revision, from previously described definitions (*21*) and grouped them as follows: asymptomatic, no symptoms; mild, any number of symptoms without fever; moderate, any number of symptoms with fever (>100.4°F); severe, respiratory distress, such as chest pain or shortness of breath; and hospitalization. If a patient record only indicated symptomatic and no specific symptoms were listed, we defined symptom severity as mild.

### Laboratory Testing

We identified all SARS-CoV-2–positive specimens by PCR in the epidemiology laboratory at the US Air Force School of Aerospace Medicine by using the TaqPath COVID-19 Multiplex assay (Thermo Fisher Scientific, https://www.thermofisher.com), CDC 2019-Novel Coronavirus (2019-nCoV) Real-Time RT-PCR Diagnostic Panel (https://www.cdc.gov), or the cobas SARS-CoV-2 test (Roche Diagnostics, https://diagnostics.roche.com). For a subset of SARS-CoV-2–positive specimens, we subsequently ran quantitative PCR (qPCR) to determine RNA genomic equivalence (*22*). We used the SARS-CoV-2 Research Use Only qPCR Primer & Probe Kit (Integrated DNA Technologies, https://www.idtdna.com), which targets 2 regions (N1 and N2) of the SARS-CoV-2 nucleocapsid gene and has an additional control that detects the human ribonuclease P gene. We used a standard curve consisting of 4 virus RNA concentrations (10^2^–10^5^) (*22*).

We selected specimens for sequencing if they had a qPCR cycle threshold of <30. We sequenced samples by using a 1,200-bp amplicon tiling approach (*23*). In brief, we extracted specimens and amplified 1,200-bp fragments, prepared libraries by using the Illumina Nextera XT Library Prep Kit, and then sequenced the libraries on an Illumina sequencing platform (Illumina, https://www.illumina.com) (*24*). We processed sequencing data by using the Mad River analysis pipeline (https://github.com/usafsam/mad_river_wf). We genotyped consensus genomes at 10× depth by using Nextclade software (*25*) and used the genotypes to differentiate between reinfections and continuing infections. We submitted consensus genome sequences to GenBank (accession nos. PP258063–640).

### Data Analysis

We used the infecting SARS-CoV-2 clade that was identified through sequencing to differentiate between COVID-19 reinfection and continuing infection cases; we used those case categories to examine the influence of time, demographics, and vaccination on reinfection dynamics. In addition, we sought to define symptom outcomes for confirmed reinfection cases. For statistical analysis, we performed 1-way analysis of variance to determine differences in overall symptom severity between groups. Furthermore, we performed odds ratio analyses to determine associations between symptom severity and variables, adjusting for confounders (first infection severity, age, time since previous infection, vaccination status, time since vaccination, and sex of patient).

**Figure 1:**
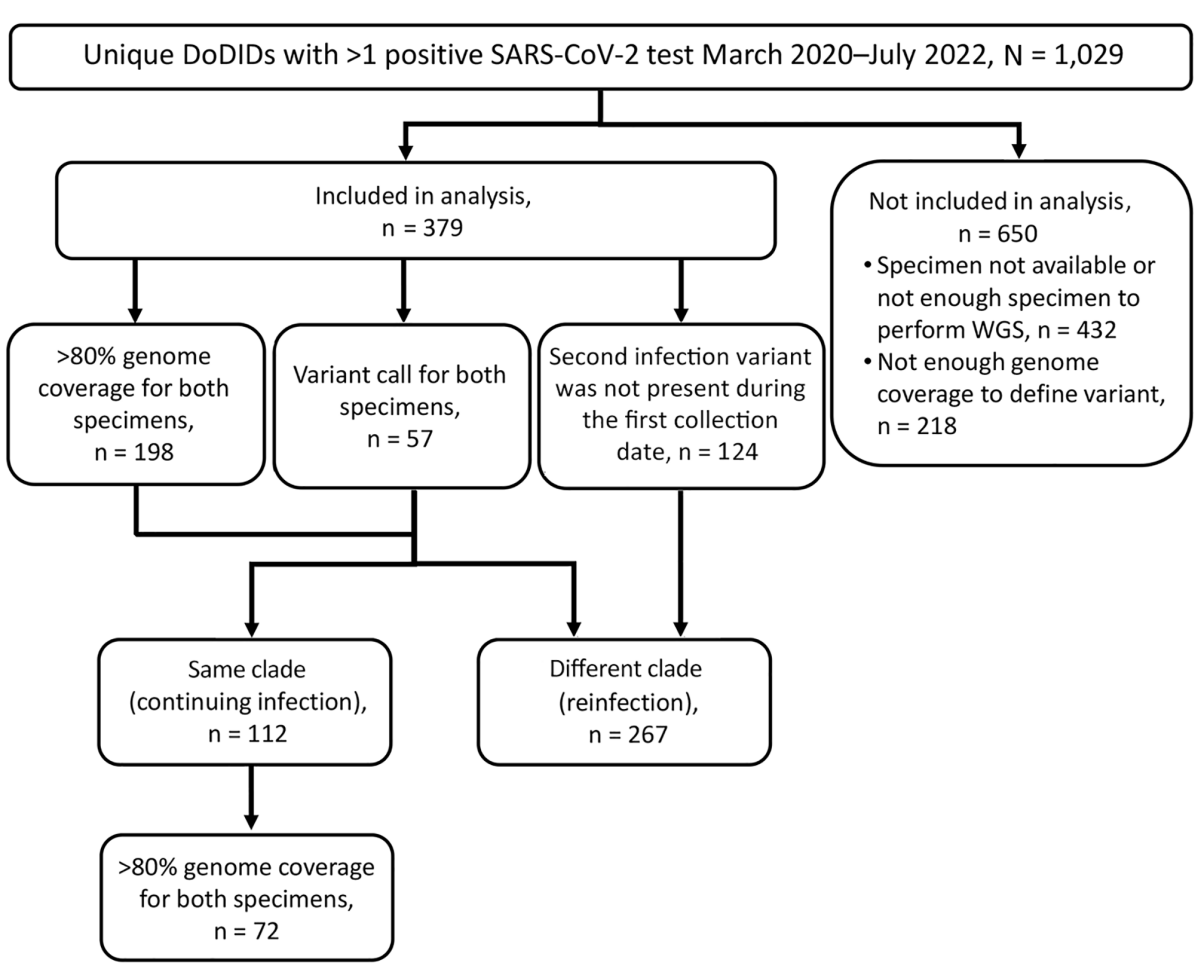
Case selection in study using SARS-CoV-2 sequencing data to identify reinfection cases in Department of Defense Global Respiratory Pathogen Surveillance Program, United States. Flowchart shows case selection criteria used to identify a SARS-CoV-2 reinfection according to whole-genome sequencing. Patient had either a reinfection if the Nextstrain (https://nextstrain.org) clade was different between the first and second specimen collection timepoints or had a continuing infection if the same clade was identified at both timepoints. DoDID, Department of Defense identification number; WGS, whole-genome sequencing.

We defined cases as patients who had >1 positive SARS-CoV-2 test during March 2020–July 2022. We categorized each case according to the following criteria: sequenced specimens from the first and second encounters had >80% of 10× genome coverage, and the clade was determined by using Nextclade; 1 or both specimens had lower genome coverage but enough coverage to determine the clade; or the clade from the first infection was unknown, but the clade from the second infection was determined and was not present during the first infection timepoint (Figure 1). Overall, if clades from the first and second collections differed, we considered this to be a reinfection. If clades were the same at the 2 collection timepoints, we considered that to be a continuing infection. 

For demographic comparisons, we matched control datasets according to the first specimen collection date; control patients had only 1 positive SARS-CoV-2 test. We performed all statistical analysis by using R version 4.2.3 (The R Project for Statistical Computing, https://www.r-project.org). We performed alignment and genomic analyses by using MEGA version 11.0.10 (https://www.megasoftware.net) and Geneious version 2023.21 (https://www.geneious.com). 

## Results

We identified 1,029 patients who had >1 positive SARS-CoV-2 test during March 2020–July 2022. After sequencing the positive specimens from those patients, we included 379 cases in our analyses. A total of 112 cases had the same virus genotype (continuing infection), whereas 267 were classified as reinfections (Figure 1). In addition, 338/379 cases were identified through residual clinical sample sequencing, whereas 41/379 were identified by a specimens collected through influenza-like illness surveillance and included questionnaires.

The number of days between the first and second specimen collection timepoints was determined for both continuing infection and the reinfection cases (Appendix Figure 1). For continuing infections, the mean number of days between collections was 9 (range 1–43) days (Appendix Figure 1), except for 2 patients who were infected with the same clade >90 days apart (Appendix Table 1). For reinfections, the number of days between the first and second infections varied; 3 reinfection cases occurred <90 days apart and were caused by the Omicron clade 21K variant (Appendix Table 2, Figure 1). We determined the timeline of collection dates for first infection and reinfection in relation to the emergence of variants of concern and important vaccine dates (Appendix Figure 2).

### Reinfections

**Figure 2:**
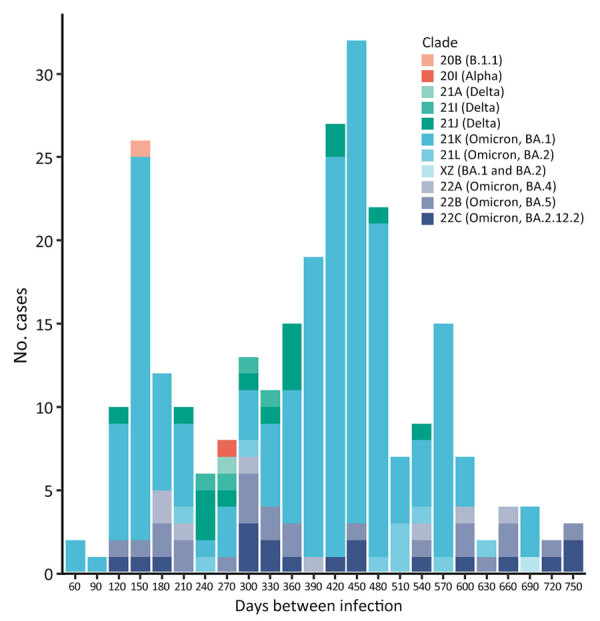
Number of SARS-CoV-2 reinfection cases in study using sequencing data in Department of Defense Global Respiratory Pathogen Surveillance Program, United States. Frequency of different SARS-CoV-2 variants relative to the number of days between the first and second specimen collection dates. One reinfection case was caused by a pre–variant of concern lineage, 1 case was a reinfection with an Alpha variant, and several cases were reinfections with a Delta variant. However, most reinfections were caused by Omicron variants. In addition, reinfections that occurred <90 days from the first infection were caused by Omicron 21K. One reinfection was caused by the XZ variant, a recombination of Omicron 21K and 21L.

We calculated the frequency of each reinfection clade according to the number of days between specimen collection dates (Figure 2). Before the Omicron variant emerged, only 9% (2/23) of reinfections occurred within 180 days of the first infection (1 each of clade 21J [Delta] and clade 20B [B.1.1]). After Omicron emerged, 21% (50/243) of reinfections occurred within 180 days. Although that difference was not statistically significant, it is consistent with the finding of an increased rate of reinfections associated with the Omicron variant, including reinfections with multiple Omicron clades (Appendix Table 3) (*26*).

We also examined an independent control dataset that was randomly matched with each first specimen collection date for reinfections, but for which patients only had 1 SARS-CoV-2–positive test. Age was the only significantly different demographic factor but was only marginally lower in the reinfection group (29.8 control vs. 27.7 reinfection; p = 0.048).

**Figure 3:**
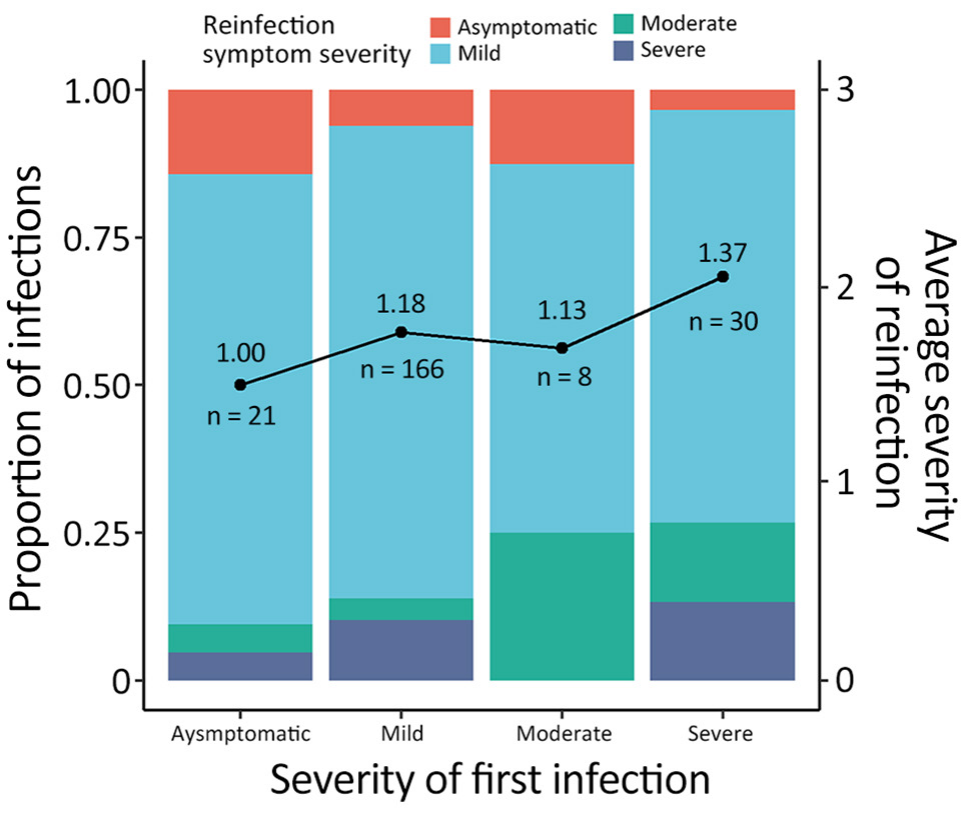
Reinfection symptom severity in study using SARS-CoV-2 sequencing data to identify reinfection cases in Department of Defense Global Respiratory Pathogen Surveillance Program, United States. Proportions of reinfections with different symptom severity at the second specimen collection timepoint are compared with the first specimen collection date. Symptom severity was assigned numeric values: 0, asymptomatic; 1, mild; 2, moderate; and 3, severe. Numbers along data line indicate the average infection symptom severity (top number) and number of reinfections (bottom number). Reinfection symptom severity correlated with symptom severity during the first infection. Relationships were determined by linear regression; adjusted p value = 0.0131, adjusted for sex and age.

**Figure 4:**
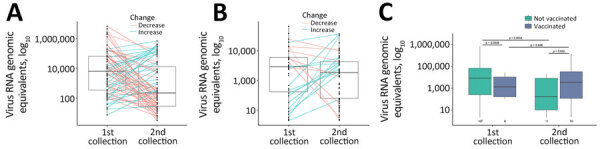
Virus load in patient specimens in study using SARS-CoV-2 sequencing data to identify reinfection cases in Department of Defense Global Respiratory Pathogen Surveillance Program, United States. Virus load was determined in specimens collected during the first and second timepoints by using quantitative PCR of the N1 region of the SARS-CoV-2 nucleocapsid gene for patients who had continuing infections (A) and reinfections (B) or who were vaccinated versus unvaccinated (C). Middle horizontal lines within each box plot are the median virus RNA genomic equivalents, outer horizontal lines indicate the interquartile range, and whiskers (vertical lines) indicate minimum and maximum data points. A) Significant decrease in virus load was observed between the first and second collection timepoints for patients who had continuing infections (p = 0.039 by Student *t*-test); average number of days between collection dates was 8.7 (range 1–43) days. B) No significant difference in virus RNA load was observed between the first and second collection points for patients who had reinfections (p = 0.290 by Student *t*-test). C) First collection group shows all first infections for patients who had either continuing or reinfections. Second collection group shows only reinfections. Numbers under box plots indicate the number of cases within each group.

In cases where symptom severity was known for both the first and second infection, we observed that patients who experienced more severe symptoms during their first infection were more likely to have greater symptom severity upon reinfection (Figure 3). No trends were observed in continuing infections (Appendix Figure 3). Furthermore, we observed that, in severe first infection cases, symptom severity increased when reinfection occurred within 6 months, compared with reinfections that occurred 12–15 (p = 0.0438) or >15 (p = 0.0366) months after the first infection (Tukey post hoc analysis controlling for vaccination status at the time of reinfection). No hospitalized case-patients were identified in this study. Female patients had greater odds of having more severe symptoms upon reinfection than did male patients (Appendix Figure 4). Age, vaccination status, time since vaccination, and time since infection did not affect the odds of increased symptom severity upon reinfection (Appendix Table 4, Figure 4). Not enough data existed to perform analyses of clade-specific effects.

### RNA Quantification

For continuing infections, we observed a decrease in the amount of virus RNA (N1 quantitation) in specimens between first and second collections; average time was 8.7 (range 1–43) days between collections (Figure 4, panel A). However, we observed no difference in the amount of virus RNA between the 2 specimen collections in reinfection cases (Figure 4, panel B). We found that vaccinated persons had significantly less virus RNA present at the time of the first infection than unvaccinated persons (Figure 4, panel C). Upon reinfection, the amount of virus RNA was significantly higher in the vaccinated group than in the unvaccinated group (Figure 4, panel C); however, most of our study population had received the COVID-19 vaccine (86% vaccinated; 1 patient received a booster dose) by that time.

### Genetic Analysis of Continuing Infections

**Figure 5:**
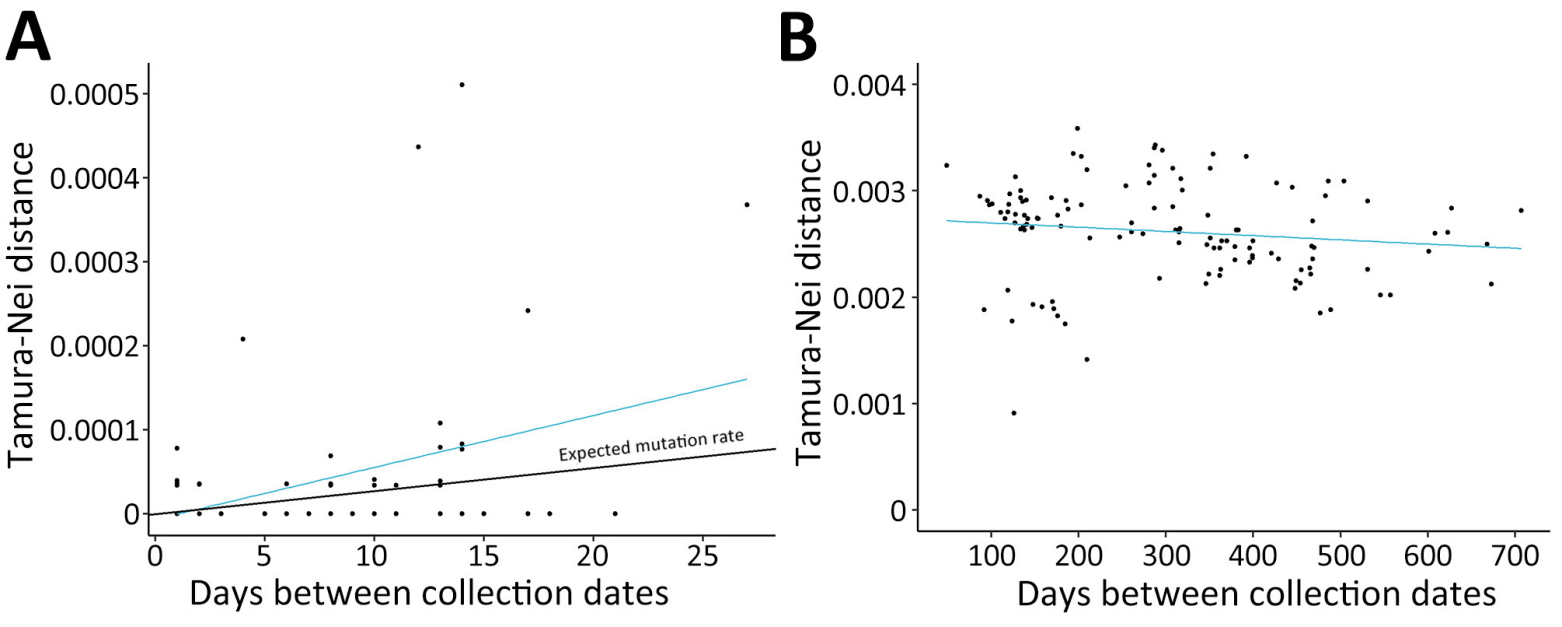
SARS-CoV-2 nucleotide changes in study using sequencing data to identify reinfection cases in Department of Defense Global Respiratory Pathogen Surveillance Program, United States. Tamura-Nei p-distances were determined relative to the number of days between specimen collection dates for continuing infections (A) and reinfections (B). A) Number of nucleotide substitutions correlated with the amount of time between specimen collections in patients who had continuing infections (p = 0.0021). Expected SARS-CoV-2 mutation rate was 1 single nucleotide variant per 2 weeks. B) No relationship was observed between number of nucleotide substitutions and time in reinfection cases (p = 0.137).

We compared sequencing data from continuing infection cases that had both the first and second collection timepoints (n = 72) to determine if nucleotide substitutions accumulated in the virus during the course of infection. The average number of days between collection dates in those cases was 7.7 (range 1–27). Using Tamura-Nei p-distance in MEGA software to quantify nucleotide changes, we found a significant relationship between the number of substitutions and time between specimen collections during continuing infections (Figure 5, panel A), which was not observed in reinfection cases (Figure 5, panel B). In addition, we saw no significant relationships between the number of nucleotide substitutions and patient sex, age, or symptom severity.

## Discussion

We leveraged SARS-CoV-2 sequencing data from the DoDGRPSP, a global DoD public health surveillance network monitoring influenza-like illness, to identify reinfection cases in the MHS. The use of this type of increasingly available public health sequencing data bolsters epidemiologic investigations pertaining to clinical manifestations of disease in patients. Although many previous studies have relied on PCR surveillance data and a 90-day threshold to define a reinfection, we show that sequencing data can differentiate between first and second SARS-CoV-2 infections by identifying variant genotypes and can also support that 90-day threshold. In addition, symptom severity during the first infection tended to predict clinical manifestations upon reinfection.

The number of SARS-CoV-2 cases in the United States increased considerably during the emergence of the Omicron variant. Many of those infections were found in persons who had already been infected with other variants and represented a substantial shift in SARS-CoV-2 epidemiology, where reinfections became commonplace (*26*). Of the 267 reinfections identified in our dataset, most occurred >90 days after the first infection; only 3 occurred under that threshold, and 2 of those 3 reinfections occurred in children (Appendix Table 2). Most reinfections in this study were caused by the 21K Omicron variant (Pangolin BA.1 lineage), which might have led to shortened time intervals between infections (*27*). As was seen for the Omicron variant, we observed an increased number of reinfections during the predominant Delta variant wave, which has been previously reported and was likely because of immune evasion over time after both vaccination and infection-acquired immunity (*3*). The amount of time needed for Omicron reinfection in this study was less than that seen for other variant waves, consistent with the shortened timeframe associated with the Omicron variant (*6*; M. Stegger et al., unpub. data, ).

Previous studies have shown that increased disease severity is expected when reinfections occur in patients <90 days from the first positive test date, particularly when the first infection was also critical or severe (*28*). Our findings support this result and suggest that, in reinfections defined by using sequencing data, symptom severity during the first infection correlated with the symptom severity during reinfection. Our findings also showed an influence of time between the first and second infections; it was more likely for patients to have increased symptom severity upon reinfection if the first infection was severe, particularly if reinfection occurred within 6 months. Our data did not have enough variability to determine differences according to the reinfecting virus clade; further investigation will be required because little is known about how different variants might contribute to SARS-CoV-2 reinfection rates.

We did not observe a relationship between vaccination status and symptom severity in reinfection cases. During the first infection, the amount of virus RNA in vaccinated persons was significantly lower than that in unvaccinated persons. However, vaccinated persons had higher amounts of virus RNA detected after reinfection than unvaccinated persons. That finding might suggest that qPCR is a poor method to determine infectious virus burden. Alternatively, the observed increase in virus RNA in vaccinated persons in this study might have been caused by immune imprinting from the initial monovalent vaccine received by the study population (*29*,*30*). Studies have shown that hybrid immunity can influence immune response upon virus reexposure (*31*).

Before the Omicron variant emerged, 1 study used a substitution rate of >1 SNV/2 weeks as a threshold to define reinfection by using SARS-CoV-2 sequencing data, observing 18 reinfections 116–342 days apart (*7*). Also, in that study, continuing infections showing substitution rates <1 SNV/2 weeks were observed >90 days apart (*7*). After the Omicron variant emerged, that same substitution rate measure was used to document reinfections involving the same Omicron clade, including some reinfections that had only 27 days between specimen collection dates (*8*). Using clade definitions to define reinfections in this study, we found many continuing infections in which the mutation rate for the SARS-CoV-2 virus was greater than expected. Accordingly, if we had used the previously reported substitution rate threshold of >1 SNV/2 weeks (*7*), 23 of our continuing infections would have been identified as reinfections, some having only a 1-day difference between collection dates. In this study, we excluded 7 cases that were inferred to not be continuing infections but were more likely co-infections by very closely related clades. Our findings highlight several considerations when using SARS-CoV-2 sequencing data to define reinfection status. Although the average mutation rate for SARS-CoV-2 viruses is 1 SNV/2 weeks, considerable interhost variation is likely because the virus interacts with more complex immune responses in populations continually exposed to emerging clades (*32*) and because patients might be immunocompromised (*33*). It will be crucial to continue investigating how emerging clades cause reinfections, which might shift our current understanding and definition of reinfection.

The first limitation of our study is that we leveraged a public health surveillance system that collects data on MHS beneficiaries who manifest influenza-like illness at clinics, as well as opportunistic sampling of SARS-CoV-2–positive specimens. Thus, this study is not a clinical observation study following persons over time, which would be a more powerful study design to assess reinfection and continuing infection dynamics. The data were collected without knowledge of prior infection history, except for those data that were captured in the medical and testing records available for public health surveillance. Using molecular testing data combined with our inability to gather symptom onset information for every case limits our ability to control for when samples were collected. Second, analysis of the military population is not generalizable because of health, age, and gender distribution limitations. Although any active-duty military, military dependent (child or spouse), or retired military member could be included in the analysis, most (66%) patients were male, and the average age was 29.7 years. The surveilled populations consisted of generally healthy persons, which limits our analysis of any underlying illnesses. Furthermore, active-duty members were required to receive a COVID-19 vaccine during this study period. Therefore, the percentage of vaccinated persons in this study (86% vaccinated by their second collection date) was significantly higher than the percentage of vaccinated persons nationwide (62%–63% in January 2022; p<0.0001 by χ^2^ test) (*34*). Vaccination reduces symptom severity (*35*), which might skew the data toward persons who have less severe symptoms.

In conclusion, we used sequencing data to differentiate SARS-CoV-2 variant genotypes and analyze infection dynamics of emerging clades in a military population. Symptom severity during the first infection tended to predict clinical severity after reinfection. Continued investigations of reinfections caused by emerging SARS-CoV-2 variants of concern by using advanced molecular methods, such as whole-genome sequencing, is needed to maintain DoD’s military readiness, and the additional clinical information gathered will benefit the general population.

Appendix Additional information for using SARS-CoV-2 sequencing data to identify reinfection cases in Department of Defense Global Respiratory Pathogen Surveillance Program, United States.
