# Supplementary material for: Using SARS-CoV-2 Sequencing Data to Identify Reinfection Cases in the Global Emerging Infections Surveillance Program, United States
Source: Emerg Infect Dis. 2024 Nov;30(Suppl 2):S53–61. doi: 10.3201/eid3014.240231 (PMC11559565; doi:10.3201/eid3014.240231)
Supplement: Appendix — Additional information for using SARS-CoV-2 sequencing data to identify reinfection cases in Department of Defense Global Respiratory Pathogen Surveillance Program, United States. [file 24-0231-Techapp-s1.pdf]

Article DOI: https://doi.org/10.3201/eid3014.240231

EID cannot ensure accessibility for supplementary materials supplied by authors. Readers who have difficulty accessing supplementary content should contact the authors for assistance.

Using SARS-CoV-2 Sequencing Data to Identify Reinfection Cases in Department of Defense Global Respiratory Pathogen Surveillance Program, United States

Appendix

Appendix Table 1. Patients who had same SARS-CoV-2 infection for >90 days

Patient no.	Age, y/sex	First collection date	Second collection date	No. days*	Variant	COVID vaccination†		Symptoms†	
						First	Second	First	Second
394	30/M	2021 Apr 8	2021 Jul 19	102	Alpha 20I	Unknown	Unknown	Asymptomatic	Unknown
360	33/M	2021 Aug 2	2021 Nov 22	112	Delta 21J	No	Yes	Mild	Mild

*Number of days between collection dates.
†Vaccination status or symptoms at time of first and second specimen collection dates.

Appendix Table 2. Patients who had SARS-CoV-2 reinfection within <90 days

Patient no.	Age, y/sex	First collection	Second collection	No. days*	Variant†		COVID vaccination†		Symptoms†	
					First	Second	First	Second	First	Second
410	31/M	2021 Nov 16	2022 Jan 6	51	Unknown	Omicron 21K	Yes	Yes	Mild	Moderate
413	11/F	2021 Nov 16	2022 Jan 4	49	Delta 21J	Omicron 21K	No	No	Severe	Severe
791	6/F	2021 Nov 16	2022 Feb 11	87	Delta 21J	Omicron 21K	Unknown	Unknown	Unknown	Mild

*Number of days between collection dates.
†Detected variant, vaccination status, or symptoms at time of first and second specimen collection dates.

Appendix Table 3. Distribution of SARS-CoV-2 clades during first infection and reinfection*

	First infection clade†													
Second infection clade (variant)	Unknown	19A	B.1.x	20A	20B	20C	20G	20I	20J	21A	21C	21I	21J	21K
19A (B)	0	0	0	0	0	0	0	0	0	0	0	0	0	0
19B	0	0	0	0	0	0	0	0	0	0	0	0	0	0
20A (B.1)	0	0	0	0	0	0	0	0	0	0	0	0	0	0
20B (B.1.1)	0	0	0	0	0	0	1	0	0	0	0	0	0	0
20C	0	0	0	0	0	0	0	0	0	0	0	0	0	0
20G (B.1.2)	0	0	0	0	0	0	0	0	0	0	0	0	0	0
20I (Alpha)	0	0	1	0	0	0	0	0	0	0	0	0	0	0
20J (Gamma, P.1)	0	0	0	0	0	0	0	0	0	0	0	0	0	0
21A (Delta)	0	0	1	0	0	0	0	0	0	0	0	0	0	0
21C (Epsilon, B.1.427/429)	0	0	0	0	0	0	0	0	0	0	0	0	0	0
21I (Delta)	0	0	2	2	0	0	0	0	0	0	0	0	0	0
21J (Delta)	0	0	14	0	0	0	1	1	0	0	0	0	0	0
21K (Omicron, BA.1)	5	1	75	8	3	10	28	6	1	2	3	5	37	0
21L (Omicron, BA.2)	0	0	3	1	0	0	3	0	0	0	0	0	3	0
22A (Omicron, BA.4)	0	0	1	0	0	0	2	1	0	0	0	1	1	2
22B (Omicron, BA.5)	0	0	6	2	0	0	0	1	0	0	0	1	8	5
22C (Omicron, BA.2.12.2)	0	0	4	0	0	1	2	1	1	0	1	0	5	3
XZ (recombinant)	0	0	0	0	0	1	0	0	0	0	0	0	0	0

*Green highlighted cells indicate the specific combination occurred ≥5 times.
†For cases where the first infection clade was unknown but the second infection clade was not present during the first collection date, the outbreakinfo R package (https://outbreak-info.github.io/R-outbreak-info) was used to define unknown first infections according to collection date and location (US state). Some unknowns remained because multiple variants were circulating at the time of collection; many were not defined beyond B.1 and were classified as B.1.x.

Appendix Table 4. Demographic information and symptom severity for patients who had reinfections*

		First infection symptom severity				Reinfection symptom severity			
Patient characteristics	All cases	Asymptomatic	Mild	Moderate	Severe	Asymptomatic	Mild	Moderate	Severe
Total no. patients	267	23	184	9	33	15	181	14	28
Age, y, mean (SD)	27.7 (9.6)	27.3 (9.4)	27.8 (9.2)	25.7 (9.6)	29.6 (9.9)	28.5 (10.6)	28.5 (9.3)	30.8 (13.8)	27.9 (8.1)
Age, y, median	25.3	25.4	25.1	27.3	29.0	28.3	26.4	27.7	25.2
Sex									
M	175	18	113	6	23	12	119	10	14
F	92	5	71	3	10	3	62	4	14
Vaccination status, first infection									
Vaccinated	23	1	14	1	4	3	12	2	5
Unvaccinated	232	21	165	7	29	11	165	11	23
Unknown	12	1	5	1	0	1	4	1	0
Vaccination status, reinfection									
Vaccinated	205	NA	NA	NA	NA	11	144	7	23
Unvaccinated	34	NA	NA	NA	NA	1	22	3	5
Unknown	28	NA	NA	NA	NA	0	15	4	0

*Values are no. patients, except as indicated. Some symptom severity data are missing; therefore, the sums of the number of cases at each infection timepoint might not equal the total number of all cases. NA, not applicable.

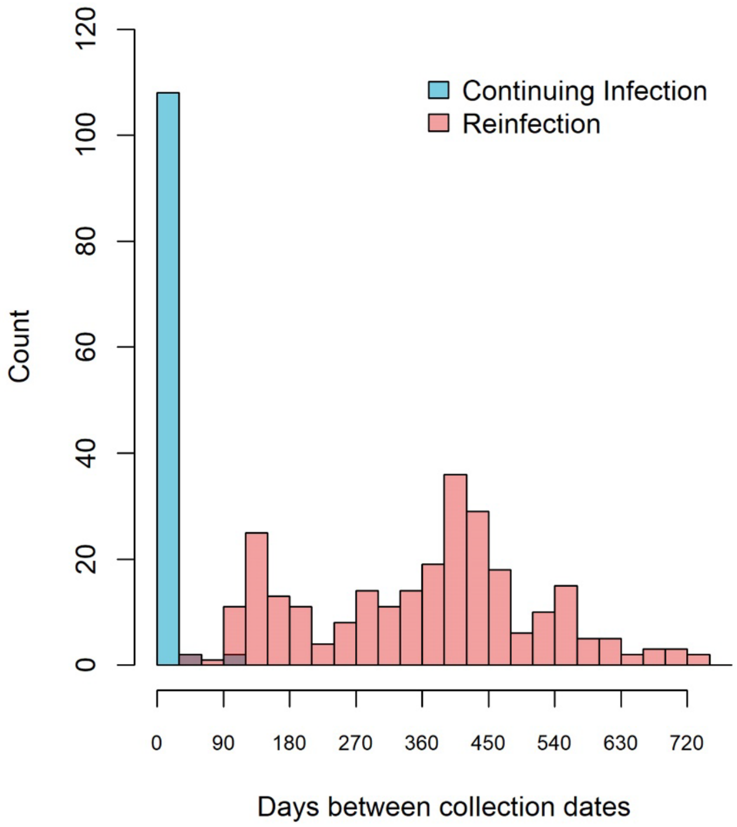

Appendix Figure 1. Distribution of the number of days between collection dates for patients who had SARS-CoV-2 continuing infections or reinfections. Patient had a continuing infection if the same virus clade was identified at both timepoints and had a reinfection if the clade was different between the first and second specimen collection timepoints. Continuing infections were primarily <30 days; however, 2 were >90 days. Most reinfections were >90 days; however, 3 occurred within <90 days. Range of days between collection dates for reinfections was 49–780 days.

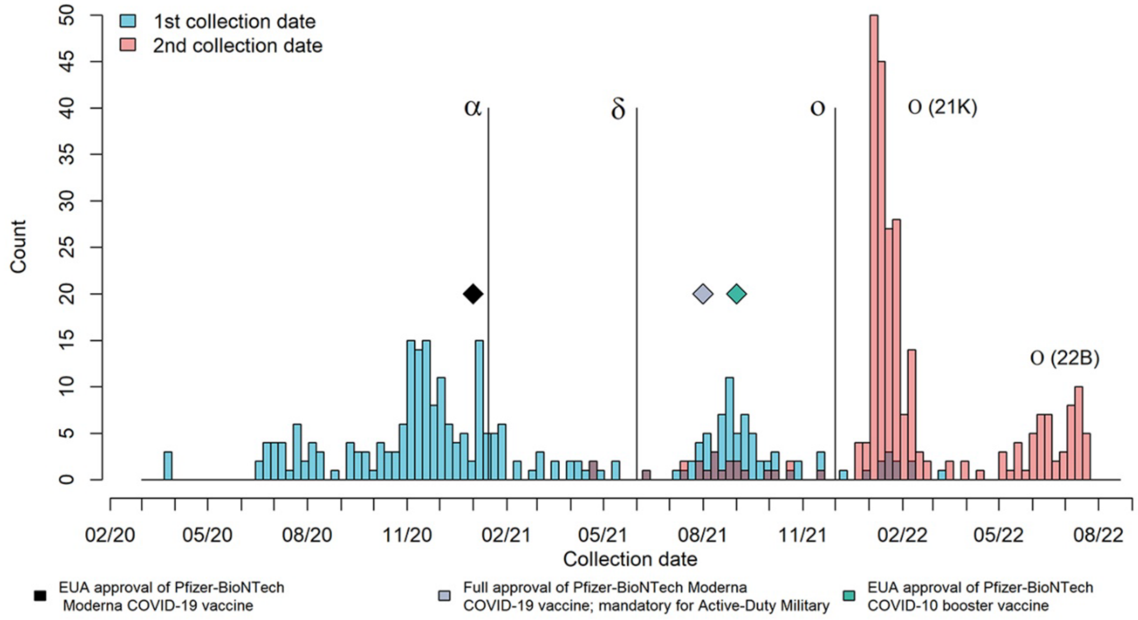

Appendix Figure 2. Distribution of collection dates for SARS-CoV-2 reinfection cases. Dates on the x axis are month/year. Blue indicates the first infection and red indicates reinfection cases. Counts are number of cases. Approximate time that variants of concern (Alpha, Beta, and Omicron) became predominant in the United States is indicated by vertical lines. Colored diamonds indicate the approval dates for vaccines and the date the vaccine became mandatory for active-duty military personnel. EUA, emergency use authorization.

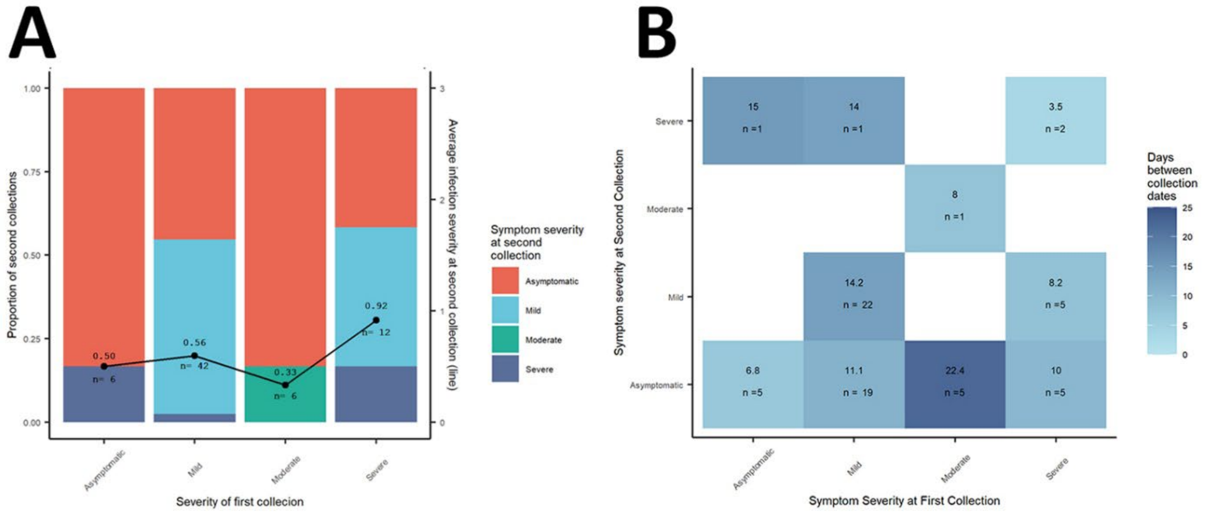

Appendix Figure 3. Symptom severity of continuing infections at the first and second specimen collection timepoints. A) Proportions of cases with different symptom severity at the second collection timepoint. Symptom severity was assigned numeric values: 0, asymptomatic; 1, mild; 2, moderate; and 3, severe.

Line indicates the average infection severity (top number) at the second timepoint and number of continuing infections (bottom number). Symptom severity at the second collection timepoint did not correlate with the symptom severity at the first collection point. B) Relationship of symptom severity between the first and second collection timepoints. The color gradient on the right indicates the average number of days between collection dates. Top numbers in the square boxes are actual number of days and the bottom numbers are the number of occurrences in each severity group.

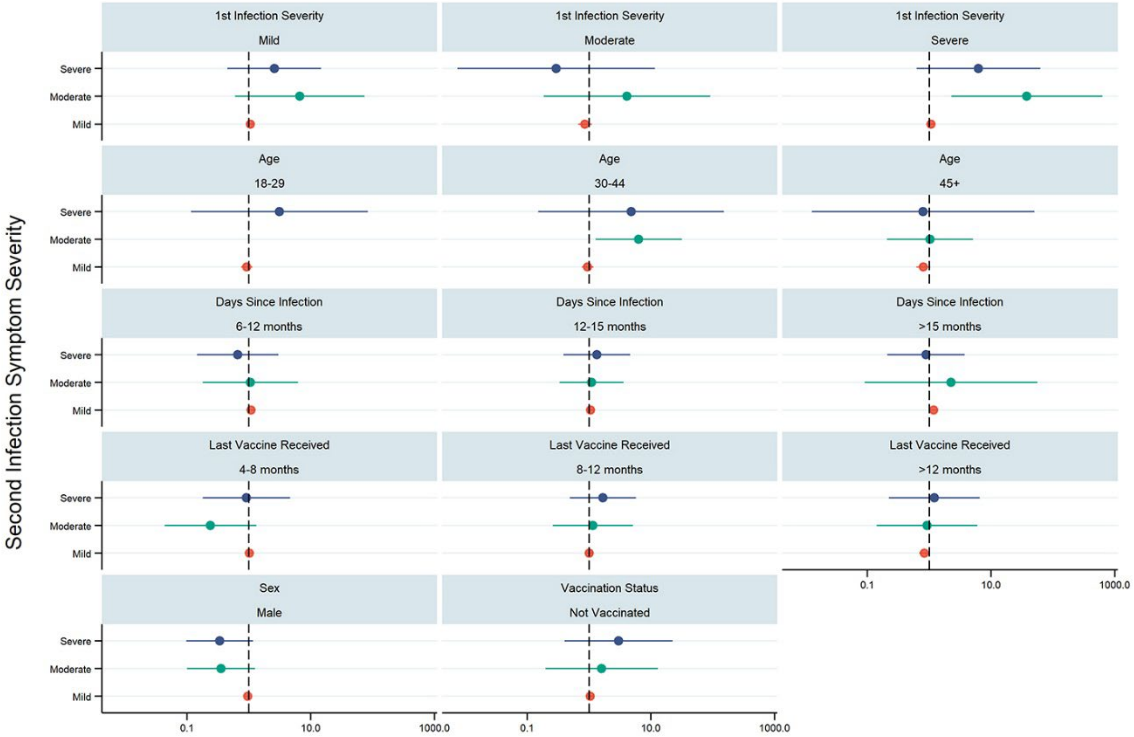

Appendix Figure 4. Odds of having more severe symptoms upon reinfection. Logistic model was run separately for each symptom classification (asymptomatic versus severe, moderate, or mild symptoms). Odds ratios were adjusted for the indicated covariates.
